# Fatal Hypermagnesemia: an acute ingestion of Epsom Salt in a patient with normal renal function

**DOI:** 10.22088/cjim.9.4.413

**Published:** 2018

**Authors:** Muhammad Shoaib Khan, Sohaib Zahid, Muhammad Ishaq

**Affiliations:** 1 Department of Cardiac MRI, Division of Cardiovascular Medicine, Allegheny General Hospital, Pittsburgh, USA.; 2 Department of Internal Medicine, Allegheny General Hospital, Pittsburgh, USA.; 3 Department of Internal Medicine, Marshfield Clinic, Marshfield, USA.

**Keywords:** psom salt, Magnesium, Hypermagnesemia, Normal Renal Functions

## Abstract

**Background:**

Generally, it is very rare for clinically significant hypermagnesemia to develop in an individual with normal renal functions as the renal handling of serum magnesium is a very potent process and it has the capacity, under conditions of hypermagnesemia, to completely block Mg (magnesium) reabsorption from the thick ascending limb of Henle. Therefore, hypermagnesemia usually arises in the setting of renal failure.

**Case presentation:**

We present a very rare case of a 40-year-old African American obese female with prior normal renal functions, who presented post-cardiac arrest following accidental overdose of Epsom salt. The patient was initially given supportive therapy and was later considered for the dialysis despite normal renal functions, as serum Mg levels kept on creeping up and clinical status kept on deteriorating continuously.

**Conclusions:**

Seemingly harmless magnesium containing (over-the-counter) (OTC) can potentially be lethal, and such consequences must always be taken into account when using such medications for a prolonged period of time

The active ingredient in Epsom salt is magnesium sulfate (MgSO4). Mg is the second most abundant intracellular cation and has essential physiological roles in the body (1). Mg has widespread therapeutic uses such as in the management of torsades de pointes, asthma exacerbation and eclampsia. However, life threatening consequences can occur if the serum Mg levels rise to toxic range. Its homeostasis is dependent on its gastrointestinal absorption and renal excretion (2). As its diffusion across the gut is mainly a passive process, therefore, kidneys play more important role in the regulation of serum magnesium by altering its reabsorption process and with normal renal functions clinically significant hypermagnesemia usually does not develop. Here we present a case of fatal hypermagnesemia following accidental overdose with Epsom salt in a patient with normal renal functions, which brings into attention the fact that a seemingly harmless magnesium containing OTC medication, can potentially be lethal.

## Case Presentation

A 40-year-old obese African American female was brought in an unconscious state to the emergency department (ED) of Allegheny General Hospital, USA, in August 2017. Patient was received at her home in a cardiac arrest state by the emergency medical services (EMS) which prompted them to do CPR (cardiopulmonary resuscitation) on her. 

With 35 minutes of CPR, spontaneous circulation was restored and then she was transferred to the hospital. On presentation, she was hypotensive, intubated, nonresponsive to noxious stimuli and had minimally reactive pupils. On further inquiry, it was found out that the patient had been ingesting Epsom salt for quite some time, apparently, to lose weight (and might have had over-ambitiously taken a large dose that day). Patient did not have any history of drug abuse or any findings suggestive of physical trauma. Differential diagnosis of acute coronary syndrome) (ACS), stroke and toxic ingestion of magnesium sulfate was contemplated. Initial blood work up revealed hypermagnesemia (9.7 mg/dL), lactic acidosis (AG metabolic acidosis), mild elevation of liver enzymes and negative urinary drug screen. 12 Lead ECG ruled out possibility of ACS, and a normal sinus rhythm with a prolonged QT (non-diagnostic for ischemia) was noted. The patient was then transferred to the ED of our hospital.

Her glasgow coma scale (GCS)at the time of presentation was 3 and ECG revealed normal sinus rhythm with first degree atrioventricular (AV) block (PR interval=220 milliseconds), QRS interval of 120 milliseconds and nonspecific intraventricular block. Brain CT scan was unremarkable for any acute intracranial process and it effectively ruled out the possibility of stroke. Despite high blood magnesium level, nephrology and poison control team initially recommended only supportive therapy without any need for dialysis as serum creatinine (0.9 mg/dL) was normal. Her urinary output was consistently normal (between 1350-1600 milliliters per day) throughout. Later, the decision was taken to dialyze the patient despite normal renal functions (creatinine=1.1 mg/dL, BUN= 17 mg/dL) because of the severity of the signs and symptoms and continuous increase of serum magnesium level in the presence of ongoing supportive therapy. The patient was then transferred to the medical intensive care unit (MICU) and it turned out that dialysis effectively lowered the serum Mg level. However, she still remained encephalopathic. Neurological assessment demonstrated bilaterally reactive pupils and extensor posturing on painful stimuli. Brain magnetic resonance imaging (MRI)findings were consistent with hypoxic-ischemic injury, possibly because of prolonged cardiac arrest, and electroencephalography (EEG) recording was also suggestive of severe cortical injury with a minimal response to pain. Meaningful recovery of the brain functions was deemed unlikely based on the clinical status of the patient, the findings of MRI and that of EEG. The family was informed about the poor prognosis of the patient and the decision was made to provide comfort measures only. Unfortunately, patient expired on the 6th post-admission day. 

## Discussion

To the best of our knowledge, only a handful of hypermagnesemia related fatal cases resulting from overdose of Epsom salt have been reported before (3, 4, 5), especially in someone with intact renal functions. Epsom salt has traditionally been considered and used for reasons including but not limited to cosmetic and therapeutic. 

The active ingredient in Epsom salt is MgSO4 . Mg is the second most abundant intracellular cation and the fourth most abundant cation in the body, which plays key roles in many functions of the body (1). Mg is an important cofactor for numerous enzymes and is crucial for the synthesis of nucleic acids and proteins. Mg homeostasis relies mainly on gastrointestinal absorption and renal excretion, and the kidney is the major organ involved in Mg regulation (2). 

Normally, the thick ascending limb of Henle’s loop has the capacity to completely block Mg reabsorption under conditions of hypermagnesemia which makes renal Mg excretion very efficient (6). Therefore, hypermagnesemia usually arises in the setting of renal failure (2). When it comes to gastrointestinal regulation of magnesium homeostasis, it is important to note that the upper small gut is the major gastrointestinal site for Mg absorption and passive diffusion is the principle regulatory mechanism (7). For that reason, massive oral Mg ingestion may result in hypermagnesemia if the absorbed amount of Mg goes beyond the renal excretory capacity (2), as it seems to be the reason in our patient.

Magnesium has long been used as a therapeutic agent for a variety of conditions and its use as a cathartic agent was its earliest therapeutic application and is being used for this purpose till date (1). It is also considered in severe asthma exacerbation where nebulized MgSO4 is considered as an addition to that with inhaled ß2-agonists (8). Its use as intravenous magnesium sulphate is recommended as the first line of therapy for torsades de pointes (9). It is also used as a prophylactic agent against the development of eclampsia in patients with severe pre-eclampsia and to prevent seizures in eclampsia patients. Nonetheless, increased serum level of magnesium can have serious consequences. Mild hypermagnesemia usually produces nonspecific signs and symptoms and may include nausea, vomiting, flushing, warmth, hypotension and lightheadedness. Magnesium concentrations of 6-12 mg/dL result in characteristic EKG changes, including prolongation of the PR interval, increased duration of QRS complex, prolonged QT interval and delayed intraventricular conduction block (10). Serum levels greater than 12 mg/dL may result in absent deep tendon reflexes, respiratory depression, paralysis, complete heart block, and at levels greater than 20 mg/dL cardiac arrest in asystole may ensue (2). Supportive therapy is the mainstay of the management of hypermagnesemia, involving respiratory support, diuresis, calcium infusion and dialysis. Calcium antagonizes the toxic effects of magnesium in addition to making up for the concomitant hypocalcemia in magnesium toxicity and, therefore, patients with severe magnesium intoxication should be given calcium gluconate. Administration of glucose and insulin also helps to promote magnesium entry into cells. Dialysis may be necessary for hypermagnesemic patients with renal dysfunction or for patients with normal renal function who have a massive overdose (11) and/or have severe signs and symptoms, as in our patient, where the decision to dialyze her was taken in the mileu of rising serum magnesium level and grave clinical status despite normal renal functions. Serum magnesium levels should be closely monitored even in patients with normal renal functions till they come back to the baseline and the precipitating factor is removed. Otherwise, higher magnesium levels may rapidly prove lethal should any complication related to the vital organs arise, as happened with this patient, where prolonged cardiac arrest resulted in irreversible diffuse injury to the brain and eventually patient died despite restoring the serum magnesium levels back to the baseline.

In conclusion fatal hypermagnesemia may ensue following acute (or chronic) overdose with Epsom salt even in people with normal renal functions. This brings into attention (yet another time) the fact that a seemingly harmless magnesium containing OTC, such as a laxative or antacid can potentially be lethal and, therefore, such consequences must not be disregarded while prescribing or using such medications for a prolonged period of time. Supportive therapy remains the mainstay of treatment of hypermagnesemia and there is no need to do urgent dialysis if the renal functions are normal, and it only needs to be considered in the setting of renal failure or deteriorating clinical status of the patient.
